# Medical Specialities

**Published:** 1854-01

**Authors:** 


### EDITORIAL DEPARTMENT.

---

*Medical Specialities.*—We have seen in the New York Daily Times, an article on this subject, discussing it fairly enough, and with considerable ability. To whom to ascribe its authorship we do not know, but presume it should be credited to the medical editor of that very able daily. The fact that a daily paper *has* a medical editor, is one of the signs of the times, to which we always mentally refer, when we see or hear those Jeremiads on the fallen condition of the profession, which are so frequent.

That we may afford our readers some notion of the article to which we refer, we quote from it at considerable length—cutting out from it, as it were, the heart of its argument.

"The student should indeed be posted in all the facts of the text-books of every chair. The applicant for a diploma, however skillful he may be in surgery, should be driven back to the ranks until he knows the principles and practice of medicine as well, else he will be merely a skillful butcher. Or, though he have great facility in prescribing, and be a marvel in diagnosis, he should be refused his "*pro auctoritate mihi commissa,*" until he is equally familiar with anatomy, and able always to trace back his path to plain English, from among the mazes of the chemical symbols. But to require that he should keep each of those collateral sciences marching on with equal pace, when circumstances have shut him up to the treatment of a narrow circle of diseases, is simply absurd. If he is dealing mostly with coughs, let him devote himself to the investigation of such troubles with his whole heart. He knows enough of chemistry to analyze the sputa—let him forswear, if need be, all chemistry that does not throw some light on his speciality. If retention is not easy, let him allow the crooked-named bones which seem like mere surplusage in the bony system, and the origin and geography of the nerves, on which ambitious students so much pride themselves, to slide into oblivion. Men who thus act may not be in perfect order to compete for all chairs in a Medical School successively, but they will make monographs and books worth reading. They keep their burning-glass fixed long enough to melt out the difficulties of the object placed in its focus. They gather upon one point heat-rays enough to reduce compounds to simples, and dissolve out the non-essentials. Men who make the eye and its diseases their sole study, must come at last to know more of its normal and morbid states, than if they had spent much of their time in tooth-pulling, and much more in watching the crises of fevers and the pointing of abscesses. If they study the eye out of its connections with the whole animal economy, of course they are not better than tinkers. We are as ignorant about the diseases of the ear, and the cures of deafness, as we were only excusable for being, a century since; and it is much because the profession frowns on its members who meditate a special attention to the ear and its ailments. So quackery has been allowed to poach on this manor, and bag all the finest game. The best treatises on uterine disorders come to us from men who spend their lives in hospitals devoted to the relief of such, and are daily familiar with the speculum.. Dentistry was a subordinate art until medical men who loved it, stopped prescribing for general disorders, and, devoting all their attention to the care and cure of diseased teeth, developed a noble science for the relief of suffering humanity."

After the portion which we have quoted follows a lively description of the numerous functions, (some of them "vicarious,") which are included in the daily routine of the country doctor. As the writer states the case, they are multiform enough. We can recall the memories of a certain day in our own practice, when we commenced with a post-mortem, and during the day reduced two broken thighs, removed one testicle, dressed a lacerated wound, pulled a tooth, attended an obstetric case, prescribed for syphilis, scarlatina, erysipelas, and ulcer of the cornea, and finally, for a case of poisoning by corrosive sublimate. Whether any one head has available knowledge enough to do all these things *well* at a moment's notice, our modesty forbids us to say.

But there is a question where no such barrier to discussion exists. Could any man do any one of these things well, without such general knowledge as in some measure qualified him for the whole? Take, for instance, the case of poisoning. When we saw it, it had continued a week, had been treated by calomel and Dover's powder, and, of course, was moribund. We will, for the sake of argument, leave out the latter condition, and suppose that the patient was not moribund, and that a mere toxicologist was called. He would find that the poison had left the stomach, and was generally diffused throughout the system. He could not give an antidote, because the stomach was in a very active acute gastritis. Call, then, the doctor who takes the stomach for his specialty. Suppose that he has the skill to cure the gastritis; then is there a mercurial tremor left, and a half paralytic condition. The poor patient (an Irish girl at service, at six shillings per week, wages) must now call in the aid of him who practices in the diseases of the nervous system—a chemist must be added to the consultation, and they must together devise a cure. We fancy that the patient would prefer the services of any single physician, always excepting the "experienced" gentleman, who prescribed originally the Hg. Chl.[1], to cure the mischief caused by Hg. Chl.[2]

Take a case of syphilis: For the chancre (we suppose that our specialist confines his practice to the disease of certain organs) he must go to Ricord, for the ulceration in his throat to Dr. Horace Green, for his rupia to the dermologist, for his corneïtis to the oculist, and to we know not whom for his node.

The advocates of specialties lose sight of the fact that an organic disease is, not unfrequently, the mere representative of a constitutional condition. It often happens that a single disease mutates in its course. In alluding to the number, or rather the variety of diseases for which we once prescribed in a single day, we purposely omitted to mention that one of the fractures, the lacerated wound, and the removal of the testicle, occurred in a single patient; while the ulcer of the cernea accompanied an erysipelas, which came on during the decline of a scarlatina.

To which of the specialties did this latter case belong? Or would it not be better to confide it to the case of a single educated physician, who was orthodox on zymosis and blood poison, and familiar with the local lesions accompanying general disease? Such instances would occur daily, and families would be much at a loss to know to whom to apply for aid.

Again the plan is certainly not applicable out of large cities, while in but few of these, could the practitioner of a specialty find business enough for his support. To do this he would be obliged to draw his patients from a surrounding region of country, and thus decrease the too slender income of country practitioners.

The last consideration we have to name is one which, while its interest is principally confined to the profession alone, would soon react upon the community.

Law, Medicine, and Divinity have long existed as the three black graces. To split up and subdivide into sections the medical profession, would destroy its influence, with its unity. Let us rather remain as one body, knit together by the ties which centuries have woven; let us maintain that organization as a republic of science which has so long endured, devoting a combined energy to the healing of infirmities; let us remain a body, strong *because united.* S. B. H.

